# Microcytosis and possible early iron deficiency in paediatric inpatients: a retrospective audit

**DOI:** 10.1186/1471-2431-9-36

**Published:** 2009-05-29

**Authors:** Deepak N Subramanian, Sarah Kitson, Amit Bhaniani

**Affiliations:** 1 Division of Medicine, ACT Health, Canberra, Australia; 2 Department of Obstetrics and Gynaecology, Addenbrooke's Hospital, Cambridge, UK; 3 Department of Public Health and Primary Care, University of Cambridge, Cambridge, UK

## Abstract

**Background:**

Iron deficiency anaemia is a common paediatric problem worldwide, with significant neurodevelopmental morbidity if left untreated. A decrease in the mean corpuscular volume (MCV) can be used as a surrogate marker for detecting early iron deficiency prior to definitive investigation and treatment. An audit cycle was therefore undertaken to evaluate and improve the identification, follow-up and treatment of abnormally low MCV results amongst the paediatric inpatients in an English district general hospital.

**Methods:**

The audit cycle was performed retrospectively over two three-month periods (February to April 2006; September to November 2006), amongst patients aged between one month and 16 years that had full blood counts performed whilst admitted on the paediatric ward. Patients with at least one abnormally low MCV result were identified, and their notes reviewed. We looked for any underlying explanation for the result, adequate documentation of the result as abnormal, and instigation of follow-up or treatment. In-between the two audit periods, the results of the first audit period were presented to the medical staff and suggestions were made for improvements in documentation and follow-up of abnormal results. The z-test was used to test for equality of proportions between the two audit samples.

**Results:**

Out of 701 inpatients across both audit periods that had full blood counts, 61 (8.7%) had a low MCV result. Only 15% of patients in each audit period had an identifiable explanation for their low MCV values. Amongst the remaining 85% with either potentially explicable or inexplicable results, there was a significant increase in documentation of results as abnormal from 25% to 91% of cases between the first and second audit periods (p = 0.00 using z-test). However, there was no accompanying increase in the proportion of patients who received follow-up or treatment for their abnormal results.

**Conclusion:**

Abnormal red cell indices that may indicate iron deficiency are frequently missed amongst paediatric inpatients. Medical staff education and the use of appropriate protocols or pathways could further improve detection and treatment rates in this setting.

## Background

Iron-deficiency anaemia in children is an important problem worldwide, estimated to affect some 43% of the world's children [1]. A study in the US quoted prevalence figures for iron deficiency of 9% for toddlers aged 1–2 years, 7% for older pre-pubescent children, 9–11% for adolescent females and 1% for adolescent males [2]. In developed countries, poor-dietary intake of iron is the commonest cause [3]. There is an association between iron deficiency and a variety of aspects of neurodevelopment in children; from reduced school achievement and behavioural problems to developmental delay [4-6].

Iron-deficiency anaemia manifests itself as a microcytic, hypochromic anaemia. Microcytosis develops either prior to or along with any reduction in haemoglobin (Hb) levels [3,7]. Hence the incidental detection of an isolated low mean corpuscular volume (MCV) result could indicate early iron deficiency that has not yet resulted in anaemia. A decrease in mean corpuscular haemoglobin (MCH), reflecting red cell hypochromia, can also be used to diagnose iron deficiency; however, since this decrease normally accompanies the reduction in MCV [8], it is no more reliable a marker than the MCV for detecting iron deficiency. With the exception of anaemia of chronic disease (ACD), other conditions that cause a microcytic, hypochromic anaemia (e.g. thalassaemia) are much rarer than iron deficiency, and other factors in the history and clinical picture will usually indicate if further testing is necessary. ACD more often causes a normocytic anaemia, but in 30–50% of cases can cause a microcytic anaemia [9]. In the context of a paediatric inpatient population, ACD is likely to be a relevant factor only in those children known to have a severe chronic illness and/or an acute, severe illness of significant duration. The majority of paediatric inpatients will have an acute, minor illness of brief duration. Previous work has suggested that although this type of illness can lead to a transient decrease in Hb, there is little or no significant change in the MCV [10,11]. This anaemia can take up to three months to resolve, depending on the level of inflammation associated with the illness [12]. There is currently no evidence that these children would have an isolated microcytosis. The red cell distribution width (RDW) can also help to differentiate ACD from iron-deficiency anaemia, since this is abnormally elevated in iron-deficiency anaemia, but not in ACD [13].

The use of the MCV as a tool for guiding selection of inpatients for further investigation of possible iron deficiency has been questioned, mainly due to its moderately poor sensitivity in detecting iron deficiency despite its apparent high specificity [14,15]. These studies however were done in a non-paediatric population, and used a cut-off point of 80 fL which may not be applicable to other population groups. One study that was performed on an outpatient population of children aged between one and six years did show much higher sensitivity and specificity values (both close to 100%) for a MCV cut-off value of 75 fL when used for detecting iron-deficiency anaemia, as opposed to iron deficiency without anaemia [16]. One suggested reasoning for this phenomenon is that very early iron deficiency develops prior to any reduction in MCV or Hb [7,8]. Occasionally, pre-analytical factors can affect the accuracy of the MCV reading by the automated red cell counter; these include auto-reactive red blood cell antibodies leading to red blood cell agglutination, and osmotic effects on red blood cells in the presence of extreme hyperglycaemia [17].

We were interested to see whether our department was adequately detecting and following up paediatric inpatients that might have iron deficiency, given the important consequences of this condition if allowed to progress to frank iron-deficiency anaemia. The department receives about 3500 inpatient admissions per year from a mixed urban and rural setting in Eastern England. Given that a substantial proportion of these patients will have blood tests, we decided to use the MCV as an indicator of whether a patient might have iron deficiency. The MCV was chosen since other tests commonly used to diagnose iron deficiency are not routinely performed in the inpatient setting at our hospital, whereas nearly every inpatient who has blood taken will have a full blood count performed and will have a MCV readily available. Further tests (including RDW, blood films, serum ferritin levels etc) might then be added to existing blood samples or performed following recovery to further characterise whether an iron-deficient state or alternative explanation for the microcytosis is present.

## Methods

The audit cycle was performed retrospectively on data from two three-month periods: February to April 2006, and September to November 2006 inclusive. Our department had a total complement of twenty-one medical staff at the time of the audits who were involved with inpatient care. All clinical decisions involving the patients had already been made prior to the commencement of data collection for each audit period.

The first period formed the basis of our initial audit; these findings were presented to our department's medical staff in May 2006. This presentation also included background information regarding iron-deficiency anaemia, the rationale for use of the MCV as a surrogate screening marker, and outlined a set of draft guidelines for medical staff regarding adequate documentation and follow up of abnormal red cell indices. These guidelines covered the use of age-specific ranges for red cell indices to better identify abnormal results, the adequate documentation of abnormal results in the notes and discharge summaries, and provided suggestions for possible follow-up tests (e.g. addition of RDW and a blood film to previous FBC tests) and other interventions (e.g. dietary advice) for those patients with borderline and significantly abnormal results. The second period formed the basis of a re-audit to see if there was any improvement in detection and follow-up rates following our presentation.

We considered all full blood count (FBC) results from the Paediatric Admission Unit and inpatient ward at our hospital (The Queen Elizabeth Hospital, King's Lynn, Norfolk, UK) from children aged one month to 16 years inclusive over the time periods specified. Requests from the Special Care Baby Unit, Outpatient Department, Emergency Department and GPs were excluded. The age-specific limits for Hb and MCV given by Nathan and Oski [18] (Table 1) were used on the results to identify those patients who had at least one FBC result with a MCV equal to or below the lower limit for their age. For those patients with more than one FBC result available, we used only the result with the lowest MCV value (or Hb value if the MCVs were equal).

**Table 1:** Normal haematological values for Haemoglobin and Mean Cell Volume in children. Adapted from [18]. Copyright Elsevier (2003).

	**Haemoglobin (g/dL)**		**Mean Cell Volume (fL)**	
**Age**	**Mean**	**Lower Limit (-2 SD)**	**Mean**	**Lower Limit (-2 SD)**

1 month	14.0	10.0	104	85

2 months	11.5	9.0	96	77

3 to 6 months	11.5	9.5	91	74

0.5 to 2 years	12.0	10.5	78	70

2 to 6 years	12.5	11.5	81	75

6 to 12 years	13.5	11.5	86	77

12 to 18 years				
Female	14.0	12.0	90	78
Male	14.5	13.0	88	78

We retrieved the notes of patients with a low MCV and identified their acute diagnoses and any chronic illnesses from detailed review of their notes. This included reviewing the notes from the admission covered by the audit period, as well as any other letters, discharge summaries, previous admission records and test results that may have been of relevance. This information was used to categorise the low MCV result as 'explicable', potentially explicable or 'explanation unknown'. 'Explicable' results were those in patients who were known to be iron-deficient already, or who had a pre-existing, long-standing chronic illness (e.g. juvenile idiopathic arthritis) that had clearly resulted in ACD with microcytosis. 'Potentially explicable' results were those in patients who had a recent or ongoing acute severe illness of significant duration, or in patients who might have undiagnosed thalassaemia based on their ethnic origin. 'Explanation unknown' results were those that could not be explained by the patient's medical history and known risk factors for other disorders.

We then determined from the notes and discharge summaries of the 'potentially explicable' and 'explanation unknown' groups whether the laboratory abnormalities (Hb and MCV) had been adequately documented as abnormal, and for those that had, whether follow-up and/or treatment was subsequently arranged for those patients. Acceptable follow up included asking the patient's General Practitioner via the discharge summary to review them with regards to their low MCV and iron status, or arranging follow-up in paediatric outpatients for this specific purpose. Acceptable treatment included dietician advice regarding iron intake, and/or the commencement of oral iron therapy.

This notes review process and categorisation of results was performed exclusively by DNS for the first audit data, and exclusively by SK for the second audit data; both investigators used the same criteria mentioned above for result categorisation and identification of information from the notes.

We used the z-test [19] to test for the equality of proportions between the two audit samples. This test is based on the assumption that the two audits are based on independent samples, and was the most appropriate test for our data. The STATA program (StataCorp, College Station, TX) was used to perform our analyses.

Since this was an internal department audit which required no extra tests or interventions to be performed on human subjects, approval from an ethics committee was not required. We confirmed this fact with the Norfolk Research Ethics Committee and hospital Research Governance Committee, prior to publication.

## Results

### First audit period (February – April 2006)

In the first audit period, 319 paediatric inpatients had at least one FBC test performed. Thirty-three (10%) had a low MCV result during their admission. The sex and age distribution for these results is summarised in Table 2.

**Table 2:** Sex and age distribution for first audit results (February – April 2006).

	**Number**	**Proportion**
Male	25	76%
Female	8	24%

<0.5 years	1	3.0%
0.5 to 2 years	1	3.0%
2 to 6 years	19	58%
6 to 12 years	9	27%
12 to 18 years	3	9.0%

In term of explicability, five patients (15%) had 'explicable' results; thirteen patients (39%) had 'potentially explicable' results; fifteen patients (46%) had 'explanation unknown' results. Excluding the patients with explicable low MCV results, seven patients (25%) had their low MCV results documented as abnormal in the notes and/or discharge summary; the remaining twenty-one (75%) had no documentation of their abnormal results and did not receive any subsequent treatment or follow-up. Of the seven patients whose results had been documented, four received iron deficiency treatment and/or follow-up; the other three received no treatment or follow-up. Overall, only four out of thirty-three patients with a low MCV (12%) received treatment and/or follow up.

### Second audit period (September – November 2006)

In the second audit period, 382 paediatric inpatients had at least one FBC performed. Twenty-eight (7.3%, as compared to 10% from first audit, p = 0.080) had a low MCV result during their admission. The sex and age distribution for these results is summarised in Table 3. Two of these patients also had a FBC result with a low MCV during the first audit period.

**Table 3:** Sex and age distribution for second audit results (September – November 2006).

	**Number**	**Proportion**
Male	15	54%
Female	13	46%

<0.5 years	1	3.6%
0.5 to 2 years	4	14%
2 to 6 years	10	36%
6 to 12 years	9	32%
12 to 18 years	4	14%

The notes of one patient with a low MCV result were unavailable for review. Of the remaining twenty-seven patients, four (15%; as compared to 15% from first audit, p = 0.49) had 'explicable' results, seven (39%; as compared to 26% from first audit, p = 0.14) had 'potentially explicable' results, and sixteen (59%; as compared to 46% from first audit, p = 0.14) had 'explanation unknown' results. One patient each in the 'explicable' and 'potentially explicable' groups also had a low MCV result in the first audit period, and had results that were similarly categorised back then as well. Excluding the patients with explicable low MCV results, twenty-one patients (91%; as compared to 25% from first audit, p = 0.00) had their low MCV results documented as abnormal in the notes and/or discharge summary; the remaining two (8.7%) had no documentation of their abnormal results and did not receive any subsequent treatment or follow-up. Of the twenty-one patients whose results had been documented, two (9.5%; as compared to 57% from first audit, p = 0.0039) received iron-deficiency treatment and/or follow-up; the other nineteen (90%) received no treatment or follow-up. Overall, only two out of twenty-seven patients with a low MCV (7.4%; as compared to 12% from first audit, p = 0.27) received treatment and/or follow-up.

**Figure 1:**
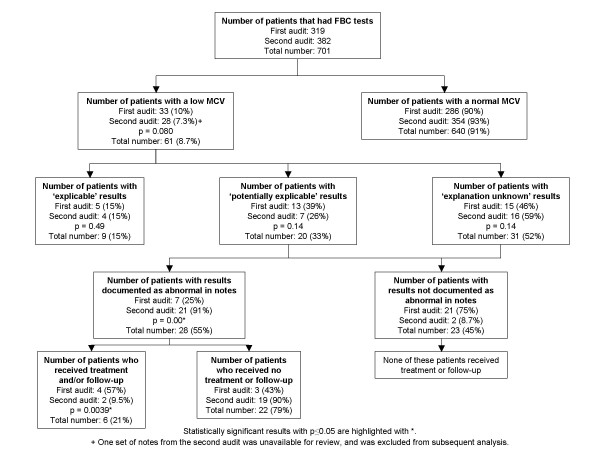
**Results of first audit (February – April 2006) and second audit (September – November 2006)**. P values are included where appropriate. All proportions and p values are given to 2 significant figures.

**Figure 2:**
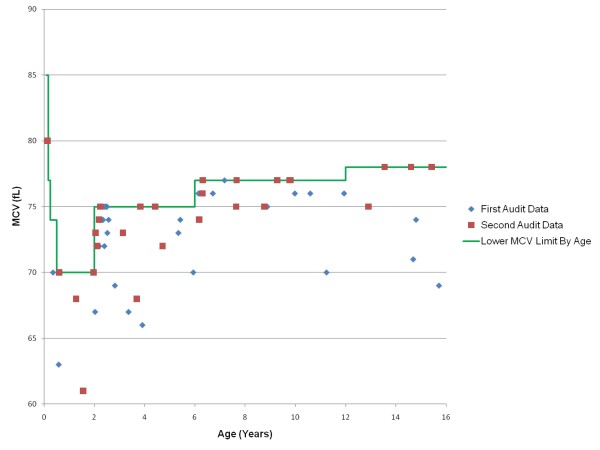
**Scatter plot of MCV results versus age**. The age-adjusted MCV lower limit (sourced from Table 1) is shown for comparison as a green line.

The overall results from both audit periods are summarised in a flow chart (Figure 1). A scatter-plot of the MCV results from both audit periods versus age, and as compared against the lower MCV limit for each age group, is also shown (Figure 2).

## Discussion

Microcytosis was not an uncommon finding amongst the paediatric inpatients who had blood tests performed; overall, 61 out of 701 inpatients (8.7%) that had FBCs performed across both audit periods had microcytosis. 30 of these patients (49%) had MCV values that were 2 fL or more below the age-adjusted lower MCV limit. The proportion of patients identified as having microcytosis that was 'explicable' remained consistent across both audits at around 15%. Since these patients' results could be clearly explained, they would not require any additional follow-up or treatment, other than that already arranged for their known illnesses.

The remaining 85% in each audit had microcytosis that was either 'potentially explicable' or 'explanation unknown'. Both groups of patients might have had iron deficiency (particularly in the 'explanation unknown' group), but diagnosis would require further dietary history from the parents and blood tests from the patients once well in order to confirm or rule out this possibility. Further testing might also reveal other disorders associated with microcytosis, such as thalassaemia. However, this must be balanced against having to do a large number of potentially unnecessary and unpleasant blood tests in order to detect the relatively small proportion of children that have true iron deficiency.

Our audit cycle demonstrated a significant and substantial improvement in documentation with regards to abnormal FBCs in those patients with 'potentially explicable' or 'explanation unknown' microcytosis, from 25% to 91% of abnormal results. This could be attributed to positive steps that were taken in our department following presentation of results from the first audit, consisting of education of other medical staff regarding the importance of adequate documentation of abnormal FBC results in the notes and discharge summaries.

However, the results with regard to follow-up of patients with abnormal results were more disappointing. Despite a far higher number of patients having their results recorded as abnormal, only two patients (as compared with four from the first audit) had subsequent follow-up and/or treatment during the second audit period. Although the educational presentation had covered follow-up and treatment of low MCV results, this was clearly inadequate in isolation for empowering the medical staff to act upon the increased number of abnormal results that would now be documented, without accompanying printed guidelines disseminated to the ward and to all staff. Further work would therefore be necessary to ensure that the staff improved on following up and treating these patients where appropriate, and that a formalised, printed protocol or patient pathway was in place to aid this.

One suggested protocol would be to take a dietary history and offer appropriate dietary advice to the parents of all patients with microcytosis where there is no immediate explanation, and to add a RDW and blood film analysis to any FBC samples that have been taken. Patients could then be stratified into low, moderate or high risk groups for iron deficiency. This would then be followed by GP follow-up with repeat FBC for the low risk patients; GP or paediatric outpatient follow-up, testing and treatment for the moderate risk patients; and in-hospital commencement of iron treatment with paediatric follow-up and further testing for the high risk patients. Further outpatient testing might include repeat FBC with a blood film, iron studies (including serum ferritin levels, transferrin levels, transferrin saturation and iron levels), and other tests as appropriate depending on the clinical context (e.g. haemoglobin electrophoresis if there is any likelihood of thalassaemia).

Current practice amongst our haematology lab is to report paediatric results as abnormal when compared against adult ranges only. This might lead to potentially abnormal results being missed by clinicians who do not then make the effort to check the abnormal results against the age-corrected ranges, or who are not familiar with them. A change in haematology lab practice, whereby results from patients under the age of 16 were reported against standardised age-specific ranges based on their date of birth, would avoid this.

For our audit, we were able to track all FBC results ordered from our ward for the relevant time periods through the computerised haematology results system. We are therefore confident that we included every paediatric inpatient in the age range specified with a low MCV result. Information was incomplete for only one patient from the second audit period whose notes were unobtainable. Our results are applicable only to hospital paediatric inpatients, and are not representative of the incidence, treatment and follow-up of microcytosis and iron deficiency in the community setting. The age-specific MCV limits that were used were taken from an American textbook of paediatric haematology, since no age-specific limits have been formulated by our local haematology laboratory for our population. It would have been better to use local norms. Our audit relied upon the subjective classification of illnesses in order to categorise MCV results by explicability, and hence was subject to operator bias. However, this was minimised by only having two analysers, who both used the same set of criteria for categorising results.

A study by Pusic *et al *[20] also looked at detection and follow-up rates of microcytosis, in all children aged 6–36 months presenting to a Paediatric Emergency Department. Over a four month period, 8% of children had a low MCV with no previously identified explanation. Physicians had documented either treatment or follow-up in only 35% of these children, and the researchers' educational interventions did not improve this detection rate. Our findings support this study's conclusions that physicians in paediatric hospital departments might be missing important information and potential opportunities to identify and treat iron deficiency when they consider FBC results, and that educational interventions on their own do not necessarily improve this.

## Conclusion

Our work demonstrates that a substantial improvement in the documentation rate or awareness of microcytosis by clinicians (and hence potential early iron deficiency) can be achieved through staff education. Follow-up and treatment rates of these patients remained low in our audit, but there are ways in which this could be improved, primarily involving education of medical staff and the use of a protocol or pathway. It would be interesting to see if our experience with identification and follow-up of these patients is similar at other hospitals in different settings.

## Competing interests

The authors declare that they have no competing interests.

## Authors' contributions

DNS designed the audit study, carried out collection and analysis of the first audit data and analysis of the second audit data, and was responsible for drafting of the final manuscript text. SK carried out collection of the second audit data, drafted the manuscript abstract and designed the results flow chart. AB performed statistical analysis on the data, and incorporated the relevant results and explanations into the final text. All authors read and approved the final manuscript.

## Pre-publication history

The pre-publication history for this paper can be accessed here:



## References

[B1] Moy RJ, Early AR (1999). Iron deficiency in childhood. J R Soc Med.

[B2] Looker AC, Dallman PR, Carroll MD, Gunter EW, Johnson CL (1997). Prevalence of iron deficiency in the United States. JAMA.

[B3] Bolton-Maggs P, Thomas A, McIntosh N, Helms PJ, Smyth RL (2003). Disorders of the blood and bone marrow. Forfar and Arneil's Textbook of Pediatrics.

[B4] Grantham-McGregor S, Ani C (2001). A review of studies on the effect of iron deficiency on cognitive development in children. J Nutr.

[B5] Lozoff B, Georgieff MK (2006). Iron deficiency and brain development. Semin Pediatr Neurol.

[B6] Lozoff B (2007). Iron deficiency and child development. Food Nutr Bull.

[B7] Oski FA (1993). Iron deficiency in infancy and childhood. N Engl J Med.

[B8] England JM, Ward SM, Down MC (1976). Microcytosis, anisocytosis and the red cell indices in iron deficiency. Br J Haematol.

[B9] Krantz SB (1994). Pathogenesis and treatment of the anemia of chronic disease. Am J Med Sci.

[B10] Reeves JD, Yip R, Kiley VA, Dallman PR (1984). Iron deficiency in infants: The influence of mild antecedent infection. J Pediatr.

[B11] Olivares M, Walter T, Osorio M, Chadud P, Schlesinger L (1989). Anemia of a mild viral infection: The measles vaccine as a model. Pediatrics.

[B12] Abshire TC (1996). The anemia of inflammation. A common cause of childhood anemia. Pediatr Clin North Am.

[B13] Bessman JD, Gilmer PR, Gardner FH (1983). Improved classification of anemias by MCV and RDW. Am J Clin Pathol.

[B14] Thompson WG, Meola T, Lipkin M, Freedman ML (1988). Red cell distribution width, mean corpuscular volume, and transferrin saturation in the diagnosis of iron deficiency. Arch Intern Med.

[B15] Seward SJ, Safran C, Marton KI, Robinson SH (1990). Does the mean corpuscular volume help physicians evaluate hospitalized patients with anemia?. J Gen Intern Med.

[B16] Hershko C, Bar-Or D, Gaziel Y, Naparstek E, Konijn AM, Grossowicz N, Kaufman N, Izak G (1981). Diagnosis of iron deficiency anemia in a rural population of children. Relative usefulness of serum ferritin, red cell protoporphyrin, red cell indices, and transferrin saturation determinations. Am J Clin Nutr.

[B17] Felgar RE, Ryan DH, Hoffman R, Benz Jr EJ, Shattil SJ, Furie B, Cohen HJ, Silberstein LE, McGlave P (2005). Automated analysis of blood cells. Hematology: Basic Principles and Practice.

[B18] Nathan DG, Orkin SH, Ginsburg D, Look AT (2003). Appendix 11: Normal haematological values in children. Nathan and Oski's Hematology of Infancy and Childhood.

[B19] Sincich T (1987). Statistics By Example.

[B20] Pusic MV, Dawyduk BJ, Mitchell D (2005). Opportunistic screening for iron-deficiency in 6–36 month old children presenting to the paediatric emergency department. BMC Pediatr.

